# Book Review: Bayesian Statistics for Beginners. A Step-by-Step Approach

**DOI:** 10.3389/fpsyg.2020.01017

**Published:** 2020-05-19

**Authors:** Jose D. Perezgonzalez

**Affiliations:** School of Aviation, Massey Business School, Massey University, Palmerston North, New Zealand

**Keywords:** Bayes, statistics, probability, philosophy, methodology

*Bayesian Statistics for Beginners. A Step-by-Step Approach* (Donovan and Mickey, 2019) is, perhaps, the “truest-to-title” book I have read on Bayesian inference and statistics, insofar (a) it is written for novices to probability, inference, the scientific method, and Bayesian methodology, (b) it introduces those four topics step-by-step, repeats them as needed, and emphasizes them throughout the entire book, and (c), despite the authors claiming that “this is not meant to be a course on statistics”(p. 269), the book delves into enough Bayesian statistics to last a lifetime. The most important contribution, however, is that this is a book purposely devoted to highlighting the role of Bayesian methodology and inference in the conduct of science.

The book could be divided into three main themes intertwined with technical expressions with which the reader will be proficient by book's end. A few chapters introduce the basic theme of probability (ch. 1–2) and probability functions (ch. 8–9). The bulk of the book delves into the theme of Bayesian inference and procedures, from introducing Bayes' theorem (ch. 3) and Bayesian inference (ch. 4–7), to presenting common Bayesian conjugates—beta-binomial (ch. 10), gamma-Poisson (ch. 11), and normal-normal (ch. 12, plus corresponding appendixes)—to working with typical Marko Chain Monte Carlo procedures—MCMC with the Metropolis algorithm (ch. 13–14), MCMC with the Metropolis-Hastings algorithm (ch. 15), and MCMC with Gibbs sampling (ch. 16). The last-but-not-least theme comprises three Bayesian applications—simple linear regression with MCMC with Gibbs sampling (ch. 17–18), Bayesian networks (ch. 19), and Decision trees (ch. 20).

Such spread of topics is met with superb depth within each chapter. This is a remarkably well-managed step-by-step approach aimed at teaching Bayesian statistics to novices, to the point that each chapter follows a challenge-response format to call attention to important topics and motivate progression. Each contains multitude of figures and tables in their appropriate locations in most step-by-step procedures and, furthermore, is in full-color (even in the printed version), as the authors use color-coded text to help keep track of each part of a formula—say, the posterior, likelihood, and prior—or of an MCMC trial—say, the proposal distributions for μ and τ–when working out the corresponding examples.

Such “for-beginners” didactic philosophy permeates the entire book, as the authors have created good synergies between chapters, to the point of using common examples to illustrate different Bayesian methodologies. For example, the normal probability density function is introduced in chapter 9, then referred to when introducing the normal-normal conjugate in chapter 12; this conjugate is used to work out the Maple Syrup problem, which is the same problem later worked out in chapter 16 using MCMC with Gibbs algorithm; finally, MCMC Gibbs is further extended into a simple linear regression application in chapters 17 and 18. Similar synergies exist among the binomial function, the beta-binomial conjugate, and MCMC with Metropolis-Hastings, between the gamma-Poisson conjugate and MCMC with Metropolis algorithm, and between Bayesian networks and decision trees.

The most important synergy, and the one I find the most exciting, is that this is a book that purposely highlights the role of Bayesian methodology and inference in scientific work. Indeed, the link to the ever-revolving scientific method—Hypothesis—Prediction—Data—Inference—is established quite early on, at the time of introducing Bayesian inference (ch. 4), and becomes a constant throughout the book from then on, as most examples are explicitly worked out following those same steps of the scientific method.

The authors seem to have also realized that the eBook version may have the largest use and, thus, have formatted the book to cater for such online environment, with ready links to external sources, such as Wikipedia and dictionaries of statistics (although, for some reason, none of those links work for me either when I access the eBook via my library or when I download chapters as pdf—and while links do not work with the printed book, the electronic formatting is still evident there). Furthermore, because an eBook is more cumbersome to learn from, each chapter of the book pretty much assumes that knowledge introduced in previous chapters is not necessarily going to be well-remembered when tackling new chapters, thus any relevant common material is summarized in depth in any new chapter, to the point of repeating the same content (say, the Lorax problem common to chapters 19 and 20) or going again step-by-step onto a previous example (say, the Shark Attack beta-binomial conjugate example before moving onto the same problem with MCMC with the Metropolis algorithm procedure). That is, each chapter practically functions as a stand-alone, self-sufficient, and fully-fledged reference on the particular topic it presents.

Above formatting may, however, play somewhat against the book as far as its focus on teaching statistics to beginners makes it cringy after a first reading, the challenge-response format gets tiring (and sometimes distracting, as when it poses questions which are not that important or which an average reader would probably not ask), and the redundancy of contents becomes burdensome. Amazingly, for the incredible detail the book goes into, very few errors seem apparent (I have only spotted an error in one of the gamma distributions in Fig. 11.11, in trials 8, 9, 10 of the MCMC in Table 15.7, and in the subscripts for *p*_*p*_ on page 240).

In a nutshell, although I still find *Bayesian Statistics the Fun Way* (Kurt, 2019; also Perezgonzalez, 2020) more insightful as an introductory book while also value the need for severe testing as part of the scientific method (Mayo, 2018; also Perezgonzalez et al., 2019), I also realize that *Bayesian Statistics for Beginners. A Step-by-Step Approach* may very well prove to be the foundational textbook for introducing Bayesian statistics and its role in the conduct of science—it certainly now surpasses those by Phillips's (1973) and Berry's (1996)—before moving onto more technical books such as Kruschke's (2015) *Doing Bayesian Data Analysis*, which may be needed for a good command of the programming background (R, JAGS, and Stan) required for mastering Bayesian statistical analyses.

## Author Contributions

The author confirms being the sole contributor of this work and has approved it for publication.

## Conflict of Interest

The author declares that the research was conducted in the absence of any commercial or financial relationships that could be construed as a potential conflict of interest.
